# An Unusual Extremely Distant Noncommunicating Uterine Horn with Myoma and Adenomyosis Treated with Laparoscopic Hemihysterectomy

**DOI:** 10.1155/2013/160291

**Published:** 2013-09-12

**Authors:** Michele Morelli, Roberta Venturella, Rita Mocciaro, Daniela Lico, Fulvio Zullo

**Affiliations:** Department of Obstetrics and Gynecology, Magna Graecia University of Catanzaro, Viale Europa, Loc alità Germaneto, 88100 Catanzaro, Italy

## Abstract

A 41-year-old woman referred to us with dysmenorrhea and severe pelvic pain although she was previously submitted to right laparotomic adnexectomy for ovarian endometrioma and to a subsequent operative laparoscopy for pelvic adhesions. After ultrasound examination, the patient underwent diagnostic hysteroscopy and operative laparoscopy which confirmed the clinic suspect of an unicornuate uterus. However, it was very unusual to see an extremely distanced right horn, without communication with uterus, without adnexa, and with a small myoma belonging to it. Moreover, omentum and bowel were attached to fundus of right horn and thick adhesions fixed it to rectum and right pelvic wall. Therefore, identification of anatomical structures was difficult, as it was extremely arduous to isolate the ureter, which was involved inside the adhesions surrounding the right uterine horn. Nevertheless, laparoscopic right hemihysterectomy was successfully performed and right horn was sent to our pathologist who recognized hypotrophic endometrium and adenomyosis.

## 1. Introduction

Unicornuate uterus is a rare congenital anomaly (2%–10% of all types of uterovaginal anomalies) [1] in which the partial development of the unilateral müllerian duct results in various degrees of rudimentary horn connected or not to the opposite horn. According to Buttram Jr. and Gibbons classification [2], the presence of a rudimentary cavitary horn, noncommunicating with the opposite one, represents the A1b variant by class II of the unicornuate uterus (7%–40% of unicornuate uterus with a rudimentary horn).

Some müllerian anomalies, including the rudimentary horns of unicornuate uterus, have increasingly been managed by surgical procedures avoiding laparotomy with excellent reproductive outcomes [3]. However, the experience of any centre is limited by the relative rarity of this particular malformation and for these reasons, diagnosis and management of such types of anomalies are often a challenge for the gynaecologist.

We present a case of a patient for whom diagnosis of uterine horn was missed at prior laparotomy and laparoscopy, which we successfully treated by laparoscopic unilateral hysterectomy for an extremely distant noncommunicating rudimentary fibromatous horn of uterus causing severe pelvic pain.

## 2. Case Presentation

In 2011, a 41-year-old woman referred to us with dysmenorrhea and persistent, severe pelvic pain for at least 8 years. After a spontaneous uncomplicated delivery at the age of 30, for the onset of secondary severe dysmenorrhea, she underwent laparotomic right adnexectomy for omolateral ovarian endometriosis.

According to patient's history, episodes of catamenial pelvic pain have been continued for several years, until she referred to a second different surgical team. In 2009, she was submitted to an operative laparoscopy for pelvic adhesions, but surgeons told her that, because of significant visceral adhesions, it was not possible to solve the problem given the risk of major intraoperative complications.

Two years later, she referred to us with persistent severe catamenial pelvic pain, not relieved even on medical therapy. Our first ultrasound showed a right sided double cystic mass, completely separated from the uterus, consistent with the diagnosis of infraligamentary myomas. Left adnexa, uterus, and both kidneys appeared normal.

Second ultrasound, performed immediately after an episode of pelvic pain, revealed a right sided cystic mass, filled with low level of homogenous echoes surrounded by a hyperechoic edge, joined to a smaller echoic mass, suggestive for small myoma. It was the sign of a recent hematometra occurred in the noncommunicating fibromatous right horn of the patient's unicornuate uterus, but the history of two previous surgeries without any mention of the presence of a müllerian malformation made it difficult for us to believe that the diagnosis was exactly this.

**Figure 1:**
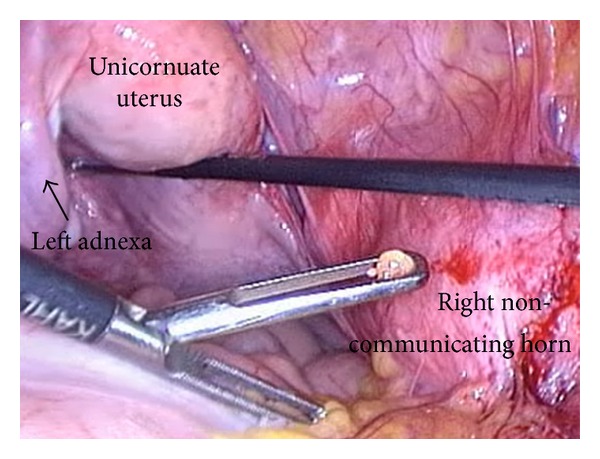
Laparoscopic vision of the completely separated and distanced right horn, with no communication with the unicornuate uterus, without any Fallopian tube or ovary, and with a small myoma belonging to it.

**Figure 2:**
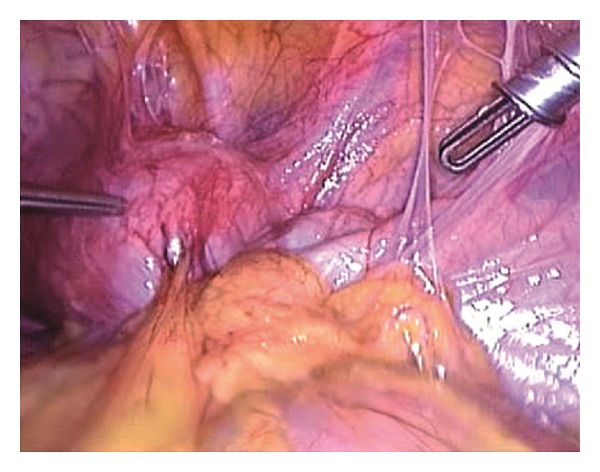
Several thick adhesion fixing the uterine horn to posterior and lateral pelvic wall.

Under general anaesthesia, the patient underwent diagnostic hysteroscopy and operative laparoscopy. Hysteroscopy revealed a slightly reduced uterine cavity with normal left tubaric ostia, normal cervix, and normal vagina. Laparoscopy confirmed the suspect of unicornuate uterus, but it was very unusual to see a completely separated and distant right horn, with no communication with the unicornuate uterus, without any Fallopian tube or ovary, and with a small myoma belonging to it (Figure 1). Moreover, probably due to previous surgeries, or related to endometriosis, many thick adhesions fixed the right horn to rectosigmoid and right pelvic wall (Figure 2). 

**Figure 3:**
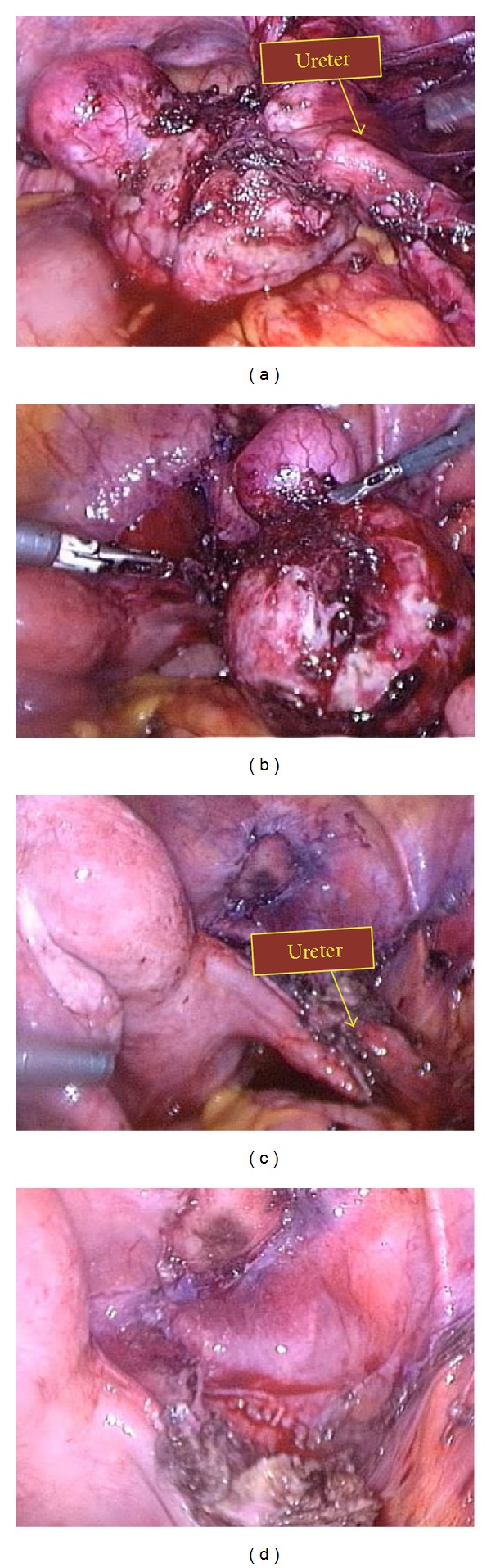
Ureteral course identification and dissection.

Lysis of omental adhesions was performed by cold scissors and blunt dissection with limited bipolar coagulation. Before starting hemihysterectomy, identification of main structures was tried but the ureteral course was not easy to recognize despite the assistance of the urologist. When he tried to cannulate the right ureteral orifice by cystoscopy, a cystic dilation of the terminal part of the ureter located within the bladder (ureterocele) was recognized. Ureteral course was finally identified by meticulous blunt dissection until it was evident that a portion of about 3 cm was involved inside the right uterine horn (Figure 3). Right round ligament and right vascular pedicle were coagulated and excised up to the uterine artery for unilateral hemihysterectomy. The right horn was morcellated and hemostasis was achieved. 

The operating time was 178 minutes and the patient was discharged on the third day. Right horn was sent to our pathologist who analyzed it and recognized myometrial tissue, hypotrophic endometrium, and focal adenomyosis. At 9 months followup, the patient feels well, and no episodes of acute or persistent pelvic pain occurred.

## 3. Discussion

The frequency of A1b variant by class II of the unicornuate uterus classified by Buttram Jr. and Gibbons is 7.7%–42.9% of all unicornuate uterus with a rudimentary horn. This unusual condition is often associated with poor reproductive function, with a greater incidence of primary infertility, pregnancy loss, and preterm labor that have been reported [4].

Nevertheless, the association with pregnancy complications is not mandatory, as shown by the obstetric history of our patient, who spontaneously gave birth to a son without any complication and who never experienced pregnancy loss.

Gynecological conditions associated with unicornuate uterus are dysmenorrhea (estimated in 70% of cases), hematometra (in 50%), and endometriosis (in 20%–40% of cases) [5]. The presence of these symptoms could help the clinician to hypnotizes the correct diagnosis, even if dysmenorrhea and endometriosis are very frequent conditions in women of reproductive age, and if hematometra does not occur, the suspect of müllerian anomaly could be omitted. For these reasons, diagnosis of unicornuate uterus is not so easy sometimes, and it can even be missed also at time of laparotomy by inexperienced surgeons.

Proper identification and diagnosis of müllerian anomaly, however, is critical to assess the right surgical approach, because the procedure is greatly influenced by the specific subtype and by anatomical characteristics of the uterus, such as the extent of the connection between the rudimentary horn and the unicornuate uterus [3].

In our case, the first ultrasonographic image of a hypoechoic mass, completely separated from the uterus, was consistent with the diagnosis of infraligamentary myomas. Confounding factors that made the preoperative identification difficult were that patient reported a history of uncomplicated pregnancy and underwent two subsequent surgeries, but nobody have still made a diagnosis of müllerian anomaly subtype. Moreover, the patient has been previously subjected to a right adnexectomy, which has made even more difficult the identification of the real nature of the masses by ultrasound. Furthermore, previous surgeries caused severe adhesions with a significant disruption of anatomical planes.

As previously described by Fedele et al. [3], the unicornuate uterus with rudimentary horn brings itself some intrinsic variations of the classic pelvic anatomy: frequently, the ureter omolateral to the rudimentary horn has a higher course, as it lies adjacent to the vascular pedicle of the horn. For this reason, it should be mandatory to identify the ureter course firstly when the round ligament is transected and the broad ligament and retroperitoneal space are entered. In our case, this surgical step was extremely complicated due to the previous surgeries which caused severe adhesions that fixed the ureteral course inside the tissue surrounding the horn.

Moreover, it has been suggested [3] to perform omolateral salpingectomy as the last surgical step, rather than excising it at the same time of the uterine horn. In our case, omolateral ovary and tube were previously excised, and also these anatomical landmarks were absent.

In conclusion, when you have an ultrasonographic pelvic pattern resembling a pedunculated infraligamentary fibroid in a patient with severe dysmenorrhea, you should always try to rule out a rudimentary horn, particularly by means of a postmenstrual ultrasound to check if an internal different ultrasonographic layer appears (small hematometra).

In managing challenging cases of anomaly, such as the one affecting our patient, surgeon skill and decision at that point of time are very important. Operative laparoscopy, with all its advantages, is a valid alternative to laparotomy, which was the approach used until now for this type of operation, but the surgical procedure can be complicated by important anatomical variations.
